# A study on Sr/Zn phytate complexes: structural properties and antimicrobial synergistic effects against *Streptococcus mutans*

**DOI:** 10.1038/s41598-022-24300-8

**Published:** 2022-11-23

**Authors:** Gerardo Asensio, Ana M. Hernández-Arriaga, Marcela Martín-del-Campo, M. Auxiliadora Prieto, Luis Rojo, Blanca Vázquez-Lasa

**Affiliations:** 1 Instituto de Ciencia y Tecnología de Polímeros, (ICTP), CSIC, C/ Juan de la Cierva, 3, 28006 Madrid, Spain; 2 Centro de Investigaciones Biológicas - Margarita Salas (CIB-Margarita Salas), CSIC, C/ Ramiro de Maeztu, 9, 28040 Madrid, Spain; 3 Facultad de Estomatología, Universidad Autónoma San Luis Potosí, Avenida Dr. Manuel Nava, 2, 78290 San Luis, México; 4 Centro de Investigación Biomédica en Red de Bioingeniería, Biomateriales y Nanomedicina, Instituto de Salud Carlos III, Madrid, Spain; 5 Interdisciplinary Platform for Sustainable Plastics Towards a Circular Economy-Spanish National Research Council (SusPlast-CSIC), Madrid, Spain

**Keywords:** Coordination chemistry, Cell death

## Abstract

Phytic acid (PA) is an abundant natural plant component that exhibits a versatility of applications benefited from its chemical structure, standing out its use as food, packing and dental additive due to its antimicrobial properties. The capacity of PA to chelate ions is also well-established and the formation and thermodynamic properties of different metallic complexes has been described. However, research studies of these compounds in terms of chemistry and biological features are still demanded in order to extend the application scope of PA complexes. The main goal of this paper is to deepen in the knowledge of the bioactive metal complexes chemistry and their bactericide activity, to extend their application in biomaterial science, specifically in oral implantology. Thus, this work presents the synthesis and structural assessment of two metallic phytate complexes bearing the bioactive cations Zn^2+^ and Sr^2+^ (ZnPhy and SrPhy respectively), along with studies on the synergic biological properties between PA and cations. Metallic phytates were synthesized in the solid-state by hydrothermal reaction leading to pure solid compounds in high yields. Their molecular formulas were C_6_H_12_0_24_P_6_Sr_4_·5H_2_O and C_6_H_12_0_24_P_6_Zn_6_·6H_2_O, as determined by ICP and HRES-TGA. The metal coordination bond of the solid complexes was further analysed by EDS, Raman, ATR-FTIR and solid ^13^C and ^31^P-NMR spectroscopies. Likewise, we evaluated the in vitro ability of the phytate compounds for inhibiting biofilm production of *Streptococcus mutans* cultures. Results indicate that all compounds significantly reduced biofilm formation (PA < SrPhy < ZnPhy), and ZnPhy even showed remarkable differences with respect to PA and SrPhy. Analysis of antimicrobial properties shows the first clues of the possible synergic effects created between PA and the corresponding cation in different cell metabolic processes. In overall, findings of this work can contribute to expand the applications of these bioactive metallic complexes in the biotechnological and biomedical fields, and they can be considered for the fabrication of anti-plaque coating systems in the dentistry field.

## Introduction

Myo-inositol hexaphosphoric acid, commonly named phytic acid (PA), is an abundant natural-occurring vitamin-B related compound that constitutes the 1–5% by dry weight of most edible legumes, cereals and seeds, and represents the major phosphorous reservoirs of plants^1^. The unique chemical structure of PA, composed of six phosphate groups that contain twelve replaceable protons, confers to the molecule a strong interaction with multivalent cations and proteins^1,2^. Moreover, the dephosphorylated species of PA by phytase enzymes play a key role in the regulation of the cellular metabolism of plants and mammalians^3,4^. In this sense, the high reactivity of PA provides a multifunctional versatility of applications in a wide range of medical treatments and daily used products since it was considered as “generally recognized as safe” (GRAS) by the Food and Drug Administration of the United States in 1997^2,3,5^. PA has been employed as a promising agent for the treatment of colonic cancer^6^ and the prevention of heart disease, renal calculi and Parkinson disease^7^; it has been implemented in several oral care products^8^, applied over metal surfaces as anticorrosion and flame-retardant coating^9^, and used as an additive for the preservation of food and packaging materials due to its capacity for inhibiting iron-catalysed oxidative reactions and microbial growth^10^.

Increasing evidence of the potential antimicrobial effect of PA has been reported in the last years. The ability of PA for inhibiting the proliferation of several foodborne bacterial pathogens^5,11^ has been described suppressing the growth of a broad spectrum of microbial species comprising gram-positive and gram-negative bacteria, some fungus, and even decreasing the production of bacterial biofilm^12–15^. The biofilm disruption ability is of particular relevance since the formation of biofilms implies the creation of a protective shell for bacteria isolating them from the host immune system and even from antibiotic treatments leading to a permanent infection^16^. These findings have attracted researches attention in order to expand the application of PA to the field of implants surface modification aiming to minimize implants failures associated with postoperative-related infections. Recently, metallic substrates have been functionalized with PA and exhibited promising results for decreasing the adhesion of gram-positive strains^17^ and titanium-PA systems also showed a bacteriostatic effect over *Porphyromonas gingivalis* cultures^18^.

Likewise, the high affinity of PA with divalent cations enables its preparation as carrier of bioactive cations in the form of metallic complexes that may tune the native properties of PA. The ability of PA to form complexes with several transition metals, alkali and alkali-hearth metals, describing the chemistry of the solution complexation equilibriums are well documented, but the molecular formulas calculated for the solid complexes are still mostly uncertain and studies lack of any type of in vitro analysis^19–22^. We hypothesise that the selective synthesis of metallic phytate complexes could modulate the bioactivity of PA regarding the cation bonded. Thus, we propose the synthesis and evaluation of two metallic derivatives of PA bearing Zn^2+^ and Sr^2+^ (named as ZnPhy and SrPhy respectively). The selected cations have been applied on regenerative therapies exhibiting a significant promotion of bone and cartilage tissues formation in vivo^23–25^. Zn^2+^ is a bioactive cation involved in several key metabolic processes and owns powerful antimicrobial, antioxidant and anti-inflammatory activities^26,27^. Besides, Zn^2+^ has been successfully incorporated on metallic surfaces or as an additive in hydroxyapatite and montmorillonite materials, exhibiting in all cases an inhibitory effect of bacterial adhesion and a reduction on the number of viable cells^27–30^. It is worth mentioning that we have recently reported the synthesis and evaluation of folic acid (vitamin B9) metallic complexes with Zn^2+^ and Sr^2+^, and found that their application as cell signalling factors provides tailored osteogenic properties in terms of ALP activity, matrix mineralization and expression of some osteogenic bone-like gens^31^. In view of all data presented, the incorporation of bioactive cations may contribute with novel properties or an enhancement of the native demonstrated potential of PA, and thus, the in vitro biological characterization of phytate complexes is of special interest for their further application in biomedicine and as food or packing additives.

Antecedents reporting spectroscopic and chemical characterization of Zn-phytate complexes in the solid-state^22,32–34^ and in the solution equilibrium chemistry^35–37^ have been described in the literature, but as far as we know, published papers on Sr-phytate complexes are scarce and in general they lack empiric data, and molecular formula in solid-state and solution complexation ability has been succinctly reported^21^. Generally speaking, it can be said that the chemistry of bioactive metallic complexes lacks empiric data, and molecular formula, either in solid-state or solution complexation, and have been succinctly reported. In this frame, the novelty of the present work lies in providing valuable knowledge in the chemistry of these complexes as well as in their biological properties, to extend their application in the biomedical field. Thus, specifically, our paper deepens in the structural chemical analysis of two bioactive phytate complexes and in their bactericide properties, and proposes their application in dental implantology.

Therefore, in this work we aim to carry out a deep physic-chemical characterization of ZnPhy and SrPhy in the solid-state, ZnPhy and SrPhy, that will provide further empirical spectroscopic data of interest, contributing as well to the assessment of their chemical structure and coordination mode, and to propose their application in the dentistry field for the fabrication of anti-plaque coating systems^11,18^. To this end, the determination of the molecular formula is carried out by ICP and HRES-TGA, and spectroscopic characterization is further analysed via ^31^P-NMR, ^13^C-NMR, ATR-FTIR and EDX spectroscopies. Furthermore, we carried out an in vitro approximation of the potential of PA, ZnPhy and SrPhy for inhibiting the production of bacterial biofilm by *Streptococcus mutans* (*S. mutans*) cultures. This property was measured through the count of viable cells and crystal violet staining. Additionally, we also analyzed the growth curves of *S. mutans* cultures obtained under phytate treatment by optical density measurements.

## Results and discussion

### Synthesis and physic-chemical characterization of phytate compounds

**Figure 1:**
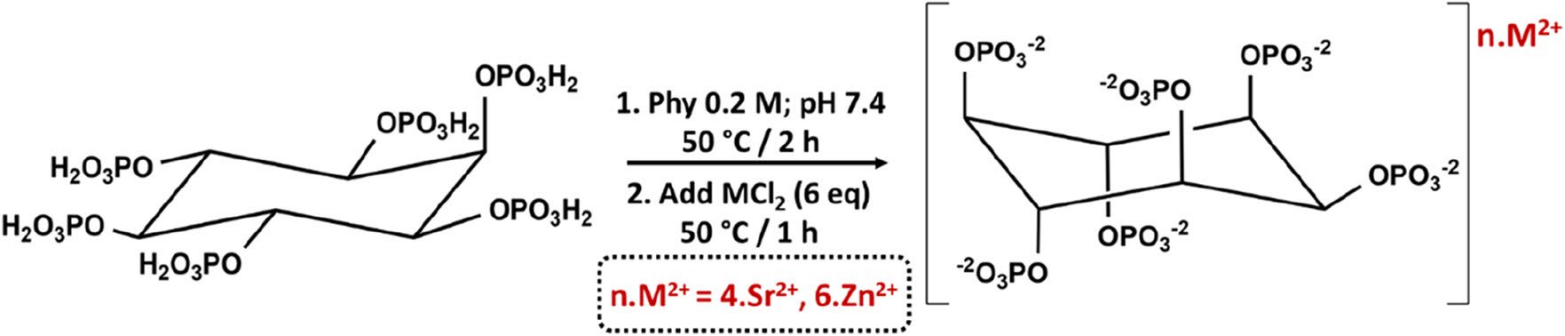
Scheme of the chemical procedure followed for the synthesis of the strontium (SrPhy) and zinc (ZnPhy) complexes of phytic acid.

The synthetic procedure followed for the preparation of SrPhy and ZnPhy derivatives is displayed in Fig. 1. Both compounds were synthesized by the hydrothermal reaction of the commercial sodium salt of PA with the respective chloride metal salt, SrCl_2_ and ZnCl_2_, and obtained in high yields (> 90%). Precipitation of metal complexes was carried out by dropping each chloride metal solution over the respective PA solutions at a pH value of 7.4 in a final proportion M^2+^:PA of 6:1. The pure phytate complexes obtained were used in their solid form for all the physic-chemical characterization. The reaction scheme showed in Fig. 1 represents a conformational change of the phytate molecule when the metallic complexes are formed as it has been previously reported by other authors^38^ and it will be discussed below in this work. Figure 1 shows a generic formula for both complexes. Nevertheless, based on reported works regarding the coordination bonds formed between the cations and the corresponding phosphate group coordination modes, and attending to the charge and the number of cations, it can be assumed that divalent cations were bonded preferably as a bridge between adjacent phosphate groups^39^.

#### Compositional analysis and thermal degradation

**Figure 2:**
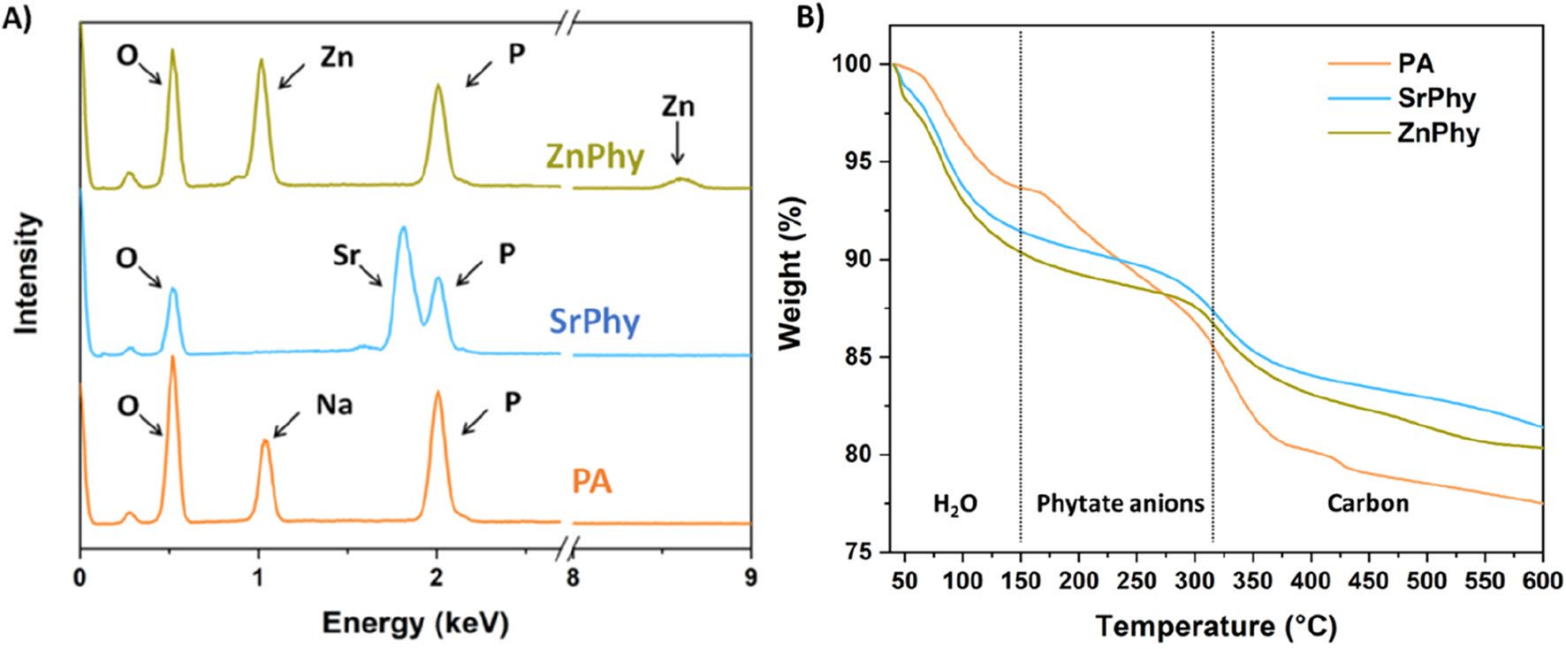
(**A**) EDS spectra registered for PA, SrPhy, and ZnPhy with assigned characteristic peaks and (**B**) HRES-TGA diagrams of PA, SrPhy and ZnPhy obtained under inert atmosphere. The dashed lines in the thermograms are shown to indicate the different regions of decomposition comprising water loss, phytate rings and organic matter.

The empiric molecular formula of the precursor and the metallic phytates are shown in Table 1. The amount of sodium, strontium and zinc coordinated to phosphate anions was quantified by ICP, and the content of water molecules was calculated from the HRES-TGA analysis. EDS spectroscopy confirmed the presence of characteristic peaks for Sr (1.80 keV), and Zn (1.01 keV), in the SrPhy and ZnPhy derivatives respectively, and for P (2.01 keV) in all compounds; EDS results also revealed that phytate complexes were obtained purely without chloride impurities, Fig. 2A. The compositional analysis of phytate complexes made by ICP and elemental microanalysis reveals that 4 and 6 metal atoms of Sr^2+^ and Zn^2+^ respectively were coordinated to phytate rings reaching P/M^2+^ molar ratios of ≈ 1.6 and 1.09 for SrPhy and ZnPhy and a P/Na^+^ molar ratio of ≈ 1 for PA. Atomic content in C and H were found as: PA 8.1%C, 2.6%H; SrPhy 6.1%C, 2.2%H; ZnPhy 6.2%C, 2.3%H. These results indicate the presence of C_6_ in the molecular formula of all compounds, and H_12_, H_10_ and H_6_ for PA, SrPhy and ZnPhy respectively. Besides, the addition of Sr^2+^ to the PA occurs in conjunction with the precipitation of Sr_4_Phy at pH 7.4 in a similar manner that has been described for Ca-Phy^40^, while Zn^2+^ forms Zn_6_Phy precipitates^22,33^.

**Table 1:** Molecular empiric formula obtained for PA and the metallic derivatives, SrPhy and ZnPhy, as determined by High Resolution Thermal Gravimetric analysis (HRES-TGA), Inductively Coupling Plasma-Atomic emission spectroscopy (ICP) and microanalysis.

Phytate compound	Molecular formula	Hydration water molecules^a^		P^b^ (% At)		Cation^b^* (% At)			C^c^ (%At)		H^c^ (%At)	
		Cal. (%)	Found (%)	Cal. (%)	Found (%)	Cal. (%)	Found (%)	P/Cation	Found (%)	Cal. (%)	Found (%)	Cal. (%)
PA	C_6_H_12_O_24_P_6_·6Na.3H_2_O	6.4	6.2	22.45	19.81	16.66	15.26	1.19	8.1	8.5	2.6	2.1
SrPhy	C_6_H_10_O_24_P_6_·4Sr.5H_2_O	8.2	8.6	16.41	16.79	31.85	30.47	1.60	6.1	6.7	2.2	1.8
ZnPhy	C_6_H_6_O_24_P_6_·6Zn.6H_2_O	9.4	9.8	16.00	16.08	34.96	32.08	1.09	6.2	6.4	2.3	1.4

^a^Determined by HRES-TGA, ^b^Phosphorous as determined by ICP, ^c^Carbon and hydrogen as determined by microanalysis *Refers to Na^+^, Sr^2+^or Zn^2+^ for PA, SrPhy or ZnPhy respectively.

When the metallic complexes were formed, the results of the HRES-TGA curves obtained in inert atmosphere exhibited different degradation profiles respect to that of PA, Fig. 2B. HRES-TGA thermograms obtained under air atmosphere are shown in Fig. S1. Up to 150 °C, PA suffered a weight loss of ≈ 5% due to the release of water molecules (T_MAX_ 92 °C). In contrast, water loss from SrPhy and ZnPhy started at early times (T_MAX_ 82 °C and 75 °C respectively) and it was faster, displaying more pronounced mass decrease compared to PA, due to their higher water content, finally resulting in 3, 5 and 6 units of water molecules coordinated to PA, SrPhy and ZnPhy structures respectively. The main degradation step has been associated with the carbonization and dehydration of hydroxyl groups^33,41^. The decomposition of phytate anions in the metal complexes (130–290 °C for both compounds) took place at lower temperature than for PA (190–380 °C). In the final degradation step, further decomposition of carbon structure occurred at ≈ 380 °C for PA and it was close to 300 °C for the phytate complexes. Once again, the presence of divalent cations coordinated with the phosphate groups produces a decrease in the thermal stability of the compounds. As expected, the residue obtained at the maximum temperature evaluated (600 °C) had a greater mass for SrPhy (80%) and ZnPhy (81%) than for PA (77%) due to the presence of the non-degradable metallic components.

Some authors have determined the complexation ability of PA with several transitions metals and found a different binding capacity attending to the phase state analysed, the solution complexation or the solid formation^19,20,22^. Overall results exhibited that under PA excess conditions, soluble species with 1:1 stoichiometry (M^2+^:P) predominated at low pH values. However, when metal cations were in excess, the precipitation of solid phytate complexes took place for which there is some diversity in the final content of water molecules and cations coordinated to phytate anion. Ermanno et al. reported that zinc-phytate compounds synthesized under cation excess conditions are composed of one water molecule, deduced by TGA, and six coordinated cations, calculated via ICP^22^. Comel et al. found that zinc complexes synthesized in the same conditions and bearing the same amount of cations per molecule, lead to six coordinated water molecules^33^, while Champagne et al. informed that Zn/phytate ratio was initially 4, and decreased to 3.5 after 24 h, monitored by^31^P NMR^32^. For the strontium complex, Gancheff et al. found a 5:1 stoichiometry between cation and phytate anion determined by elemental analysis, and owing 16 water units per molecule, for an initial mixture 5:1 (M^2+^:PA)^21^. Interestingly, the methodologies followed for the synthesis of Zn-phytate complexes employed an acidified PA solution contrary to the methodology described in this work, which may influence the coordination number of the isolated solids.

#### Raman and ATR-FTIR characterization

**Figure 3:**
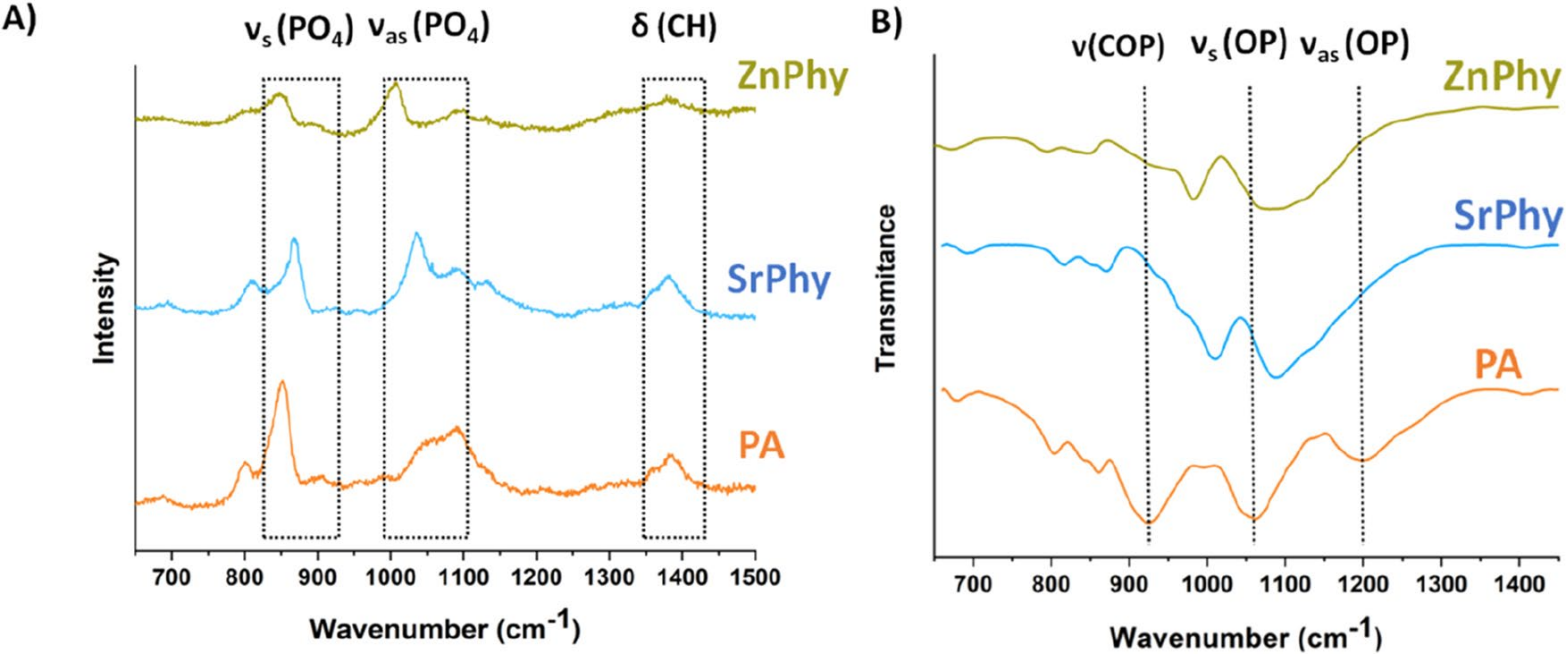
(**A**) Raman expanded spectra in the range from 650 to 1500 cm^−1^ and (**B**) ATR-FTIR spectra obtained in the range 700–1400 cm^−1^ for PA, SrPhy and ZnPhy with main vibrational bands assignment.

The conformational state 5-axial/1-equatorial of the inositol ring displayed in the reaction scheme of Fig. 1 is in agreement with the work published by Isbrandt et al.^42^. To evaluate this, Isbrant and coworkers performed a combined analysis of Raman spectroscopy, ^31^P-NMR and ^13^C-NMR with sodium phytate complexes. Raman results (Fig. 3A) have demonstrated that C–C–H and O–C–H bending vibrational bands found in the range of 1250 and 1400 cm^−1^ have a maximum intensity at 1380 cm^−1^ when PA has a 5-axial/1-equatorial arrangement, and thus, it has been assumed that all phytate compounds have the same conformation as is represented in Fig. 1. Detailed bands assignment of Raman spectra is collected in Table 2 and it is in accordance with previous reports of other authors^39,42,43^. Signals of SrPhy and ZnPhy shifted to greater or lower wavenumber when compared to those of PA bands attending to the coordinated cation. Interestingly, numerous reports have demonstrated that in the liquid equilibrium of phytic acid, the conformational state adopted below pH values of 9 corresponds to the 1-axial/5-equatorial form, conversely to the evidences obtained for the solid-state of the phytate complexes explored in this study and for other solid phytate salts^38,42^.

**Table 2:** Signal assignments of Raman spectra for PA, SrPhy and ZnPhy.

	Assignments (Wavenumber, cm^−1^)							
	Δ (CH)	ν_as_ (PO_4_)	ν_as_ (PO_4_)	Δ (OP = O)	ν_s_(PO_4_)	ν_s_(PO_4_)	ν(φ) Ring breathing + δ(COP)	γ(OH) + ν(PO_4_)
PA	1387	1084	1050	955	899	852	803	689
SrPhy	1378	1090	1007	955	892	845	808	686
ZnPhy	1383	1088	1035	954	912	867	808	694

ATR-FTIR spectroscopy analysis provided confirmation of the metal complexation of each compound. Expanded regions of the spectra obtained for each compound are shown in Fig. 3B and the assignment of the main vibrational mode bands in the whole spectra is collected in Table 3^43,44^. All compounds showed a broad band around 3300 cm^−1^ and a single peak centered in 1640 cm^−1^ which were attributed to the stretching and bending vibrational modes of O–H bonds respectively, coming either from coordinated water molecules or unbounded P–O–H bonds^39^. The main vibrational modes of the C(O)PO_3_ groups are found in the region of 750–1300 cm^−1^. The spectra obtained in this region for each compound are shown in Fig. 3B. SrPhy and ZnPhy spectra displayed peak shifts to higher wavenumber in all these bands in comparison to those of PA. This effect has been reported for similar metallic phytate complexes and it was attributed to a change in the P-O strength bond due to the modification of the chemical structure of PA in which the formation of the coordination bond takes place. The spectrum of the PA sodium salt employed in this study (C_6_H_12_O_24_P_6_·6Na.3H_2_O) exhibited a peak at 1190 cm^−1^ that corresponds to the asymmetric stretching of P–O bonds in protonated HPO^3−^ groups ^39^. Nevertheless, in the spectra obtained for the metallic compounds this band is overlapped with the symmetric stretching vibrational mode of P-O bonds. This behavior is explained by the disappearance of protonated HPO^3−^ groups due to the establishment of the coordination bond with the divalent cation^39,44^. Besides, the band centred at 917 cm^−1^ in PA spectra splits into a double peak for ZnPhy (924—980 cm^−1^), and a triplet peak in the case of SrPhy (1000–960–926 cm^−1^) as expected since the conjugation of the metal cation is different in each complex^34,39^.

**Table 3:** ATR-FTIR vibrational mode assignments of studied phytate compounds.

	Assignments (Wavenumber, cm^−1^)						
	ν(OH)– δ(OH)	δ(CH)	ν_as_(PO)	ν_s_(PO)	ν(COP)	ν(PO4)	ν(φ)ring breathing
PA	3300–1646	1396	1190	1054	917	851–835	671
SrPhy	3300–1641	1399	–	≈1250–1071	924–980	849–836	673
ZnPhy	3300–1640	1396	–	≈1250–1083	1000–960-926	861–849	683

#### ^31^P and ^13^C NMR analysis

**Figure 4:**
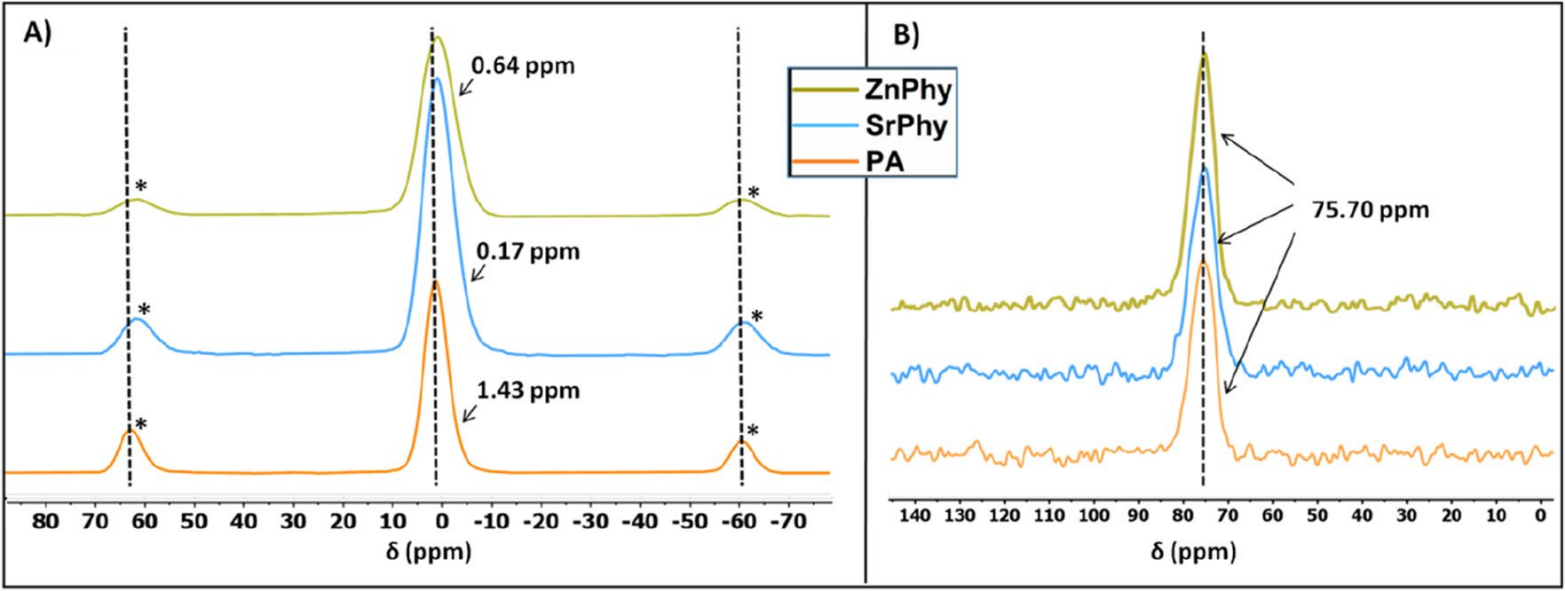
^31^NMR (**A**) and solid ^13^ NMR (**B**) spectra obtained for PA, SrPhy and ZnPhy. Spinning side bands are marked with *.

The coordination bond between zinc or strontium with phytate anion was analyzed by solid ^31^P NMR and ^13^C spectroscopy, and the results are shown in Fig. 4. For the three compounds, each ^31^P NMR spectrum presented a broad peak for all the phosphorus atoms and two symmetrical spinning sidebands (Fig. 3A). It can be observed that signals obtained for SrPhy and ZnPhy compounds moved towards lower chemical shift when compared to those of the precursor PA. This migration can be explained by the formation of a coordination bond created between Sr^2+^ or Zn^2+^, and phosphate groups, leading to a decrease in the electric dipole moment of the bridge oxygen atom, and the consequent displacement of the signals to lower chemical shifts. Similar effect has been previously reported for metallic phytate compounds in the solid state finding that both the type of metal and the number of metal-phytate bonds influence the chemical shift of the phosphorous atoms and the spinning sidebands^45^. In the solid ^13^C NMR spectra of PA, SrPhy and ZnPhy (Fig. 3B) a single peak centered at 75.7 ppm for each compounds was displayed, denoting that there is not a direct interaction between the respective metals with the carbon atoms of the molecule^42^.

In overall, at sight of the published results and those obtained in the present work regarding the structural characterization of the metallic phytates, it can be concluded that there exists a relationship between the synthetic procedure and the coordination number of the as-obtained metallic complex. The complexation ability of PA highly depends on the nature of the cation, the ratio PA:M^2+^, the pH reaction values and the ionic strength of the medium. The variability of these parameters leads to the obtaining of solid phytate complexes with different number of coordinated cations. Physical and spectroscopic data obtained for SrPhy and ZnPhy are comparable to those previously reported for similar complexes differing mainly just in the molecular formulas^33,40,43^.

### Antimicrobial activity

#### Biofilm inhibition ability

The antimicrobial potential of phytate compounds was assessed as a function of their capability to impair the growth and production of biofilm by *S. mutans* cultures. This strain has been described as one of the main cariogenic bacterial species of the oral microbiota and it is considered as an important risk factor in the development of dental caries^46–48^. *S. mutans* synthesizes glucans that promote the biofilm formation and the acidification of the buccal environment^47–52^ which leads to the proliferation of other biofilm bacterial species. Therefore, strategies to inhibit the proliferation and formation of *S. mutans* in dental plaque are key for cariogenic prevention^49,53^. In this vein, biofilm formed was studied by means of CV staining and colony forming unit counts (CFU) in agar-BHI solid plates. CV is a protein dye commonly used for the identification of all biofilms that stains the extracellular matrix of polysaccharides and negatively charged surface molecules^54^. This method implies an improvement in the determination of total biofilm and not just functional biofilm, since CV can dye both viable and dead cells together with extracellular matrix^55^. Phytate compounds were dissolved in a mixture of BHI:Tris–HCl 50 mM (1:1) at 100 µg/mL, and tested at a final concentration of 50 µg/mL. As control sample, bacteria were treated with a mixture of BHI:Tris–HCl 50 mM (1:1) emulating the same culture conditions established for the experimental samples but without phytate supplementation. The concentration of the Tris–HCl buffer employed was reported to not affect bacterial growth^56^. Moreover, based on the thermodynamics equilibriums previously established for both phytate complexes, the expected phytate species found in solution at the experiment pH value (≈7) are Zn^2+^ (83%) and ZnH_3_Phy^7−^ (17%) for Zn-containing complexes; and Sr^2+^ (≈71%) and SrH_5_Phy^5^ (≈29%)for strontium phytate compounds^21,33^.

**Figure 5:**
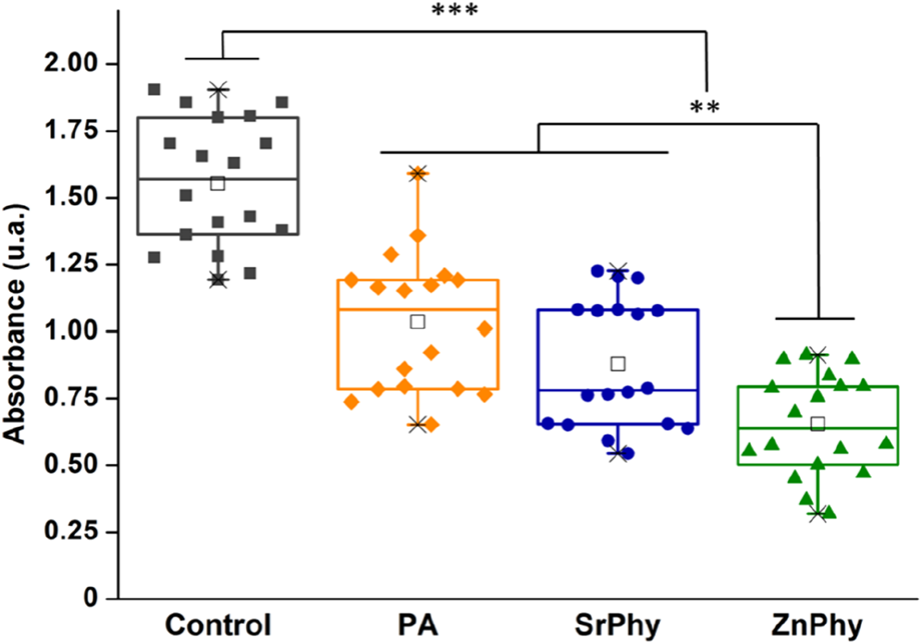
Relative inhibition capacity of biofilm production exhibited by CV staining of *S. mutans* biofilms under treatment with phytate compounds (50 µg/mL). Statistical differences between samples were analysed by ANOVA test at significant levels **p < 0.005 and ***p < 0.001 (Tukey Test).

The quantification of CV found in the biofilms formed is represented as the optical density in Fig. 5. Inhibition capacity was composition-dependent regarding the cation bonded in each PA-complex. All phytate compounds were able to significantly reduce the biofilm produced by *S. mutans* (***p < 0.001), and interestingly, cells treated with ZnPhy exhibited a remarkably improved anti-biofilm activity with respect to PA and SrPhy samples (**p < 0.005). In parallel, the count of viable bacteria found in both the biofilm matrix and the planktonic supernatant were determined. There is only a significant bactericidal effect in the resuspended biofilm matrix (nearly 1 log) for ZnPhy sample (*p < 0.005) (see Supplementary Fig. S2), which is in accordance with the biofilm disruption assessment (Fig. 5). On the contrary, the CFU counts found in the planktonic solution did not show significant differences, suggesting that the antimicrobial activity when phytate treatment was applied is mainly effective for the biofilm formation. Therefore, our results demonstrate the effectiveness of two metallic phytate-complexes bearing Sr^2+^ and Zn^2+^ (SrPhy and ZnPhy) to prevent the *S. mutans* biofilm.

However, the metabolic role of PA in the disaggregation of bacterial biofilms remains poorly understood. It is believed that the antibacterial mechanism of PA is based in the disruption of the cell membrane integrity due to the high negative charges of its chemical structure, and thus, cellular morphology and intracellular ATP levels may be affected^8,11,12^. This theory is supported by the broad spectrum of both gram-positive and gram-negative bacteria for which PA has demonstrated antibacterial potential, and also by the rapid action required to achieve antibacterial activity^15^. Another explanation of the mechanism of action could be associated with the iron-chelating properties of PA since there are some reports that support the anti-biofilm ability for other iron-chelating agents^43–45^.

The greater disaggregation of the polymeric biofilm observed for Sr/Zn-bearing phytate complexes can be understood as a possible synergic effect between the positive cation and the negative charges of PA that may hinder the aggregation of proteins required for the adhesion of biofilm-forming polysaccharides presumably by the establishment of a ternary protein-metal-phytate complex^46,47^. For its part, other authors have explored the combined effect exhibited by antibiotic-based systems including Zn^2+^. The formation of Zn^2+^ complexes with kefzol (a commercial antibiotic) has been reported to remarkably improve the antibacterial activity exhibited by kefzol treatment alone^48^. The system zinc citrate/triclosan was also analysed and a similar synergic antimicrobial effect against *S. mutans* was detected, attributed to the presence of Zn^2+^^49^, which agrees with our findings. Zinc has been reported for affecting *S. mutans* metabolism, at mM concentration, via multiple inhibitory actions comprising the modulation of oxoenzymes, the inhibition of glycolysis, alkali production, the function of the phosphoenolpyruvate system (sugar phosphotransferase, PTS) and F-ATPase^49–52^, which allow biofilm growth to be controlled. However, the antimicrobial effect of Zn^2+^ by itself seems to be bacteriostatic since the inhibition of glycolysis, PTS and F-ATPase were reversible processes^50^. Thus, Zn^2+^ is expected to only may enable bacteria killing in combination with other bactericidal agents^50^, though an improvement of their intrinsic potential was noticed as described above, and also supported by our results^48,49^. On the contrary, a recent study found equal bacteriostatic and bactericidal properties for zinc sulphate and zinc acetate salts (tested in the range of µg/mL) against *S. mutans* cultures^53^. In our work, a low concentration of Zn^2+^ (≈18 µg/mL) enabled to reduce the production of biofilm by *S. mutans.*

#### Growth curves

**Figure 6:**
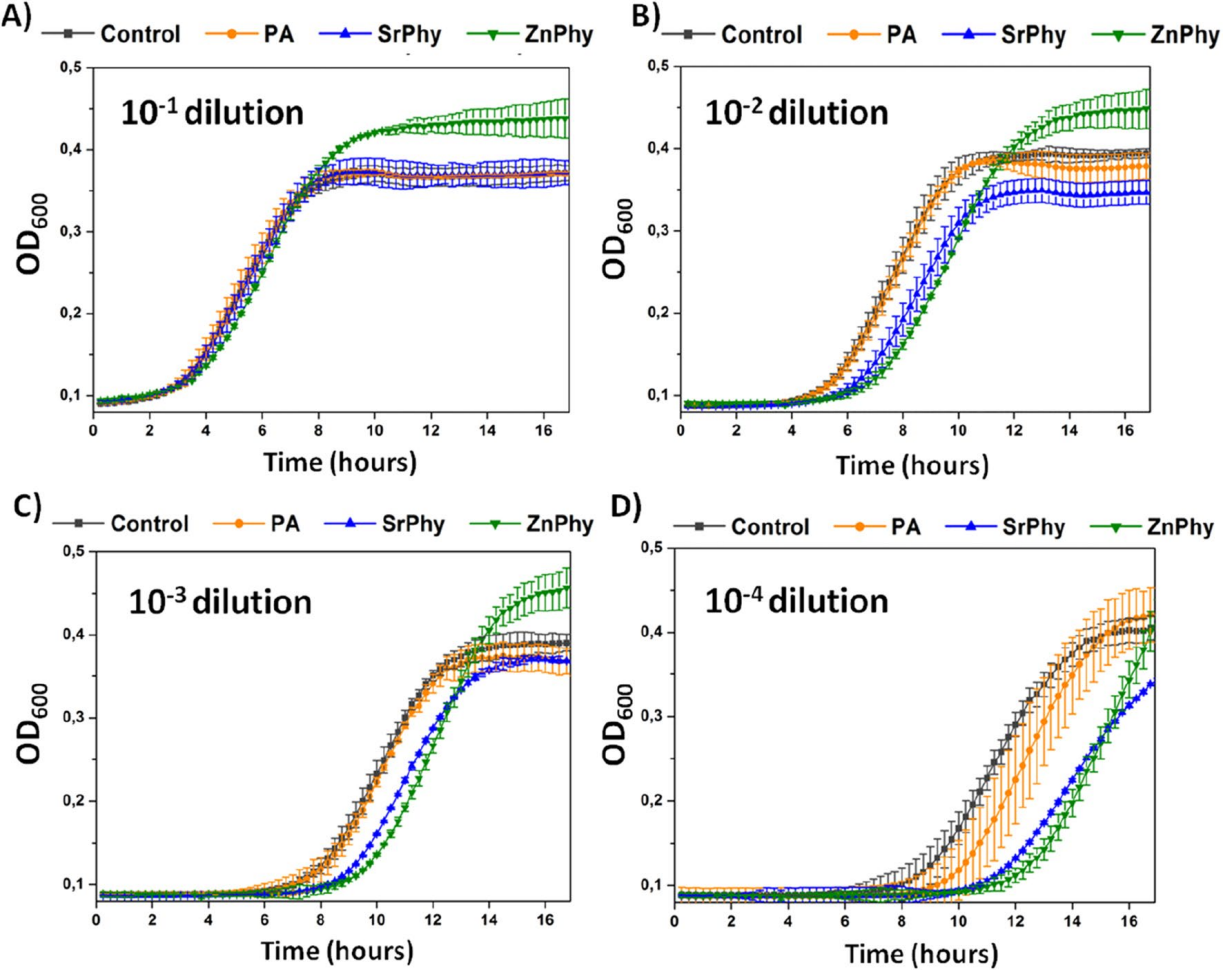
Growth curves were obtained from ten-fold serial dilutions (ranging **A**–**D** from 10^−1^ to 10^−4^) of *S. mutans* culture at OD_600_ 0.2, grown under treatments with phytate compounds (50 µg/mL). The control sample was a diluted culture growth in BHI:Tris–HCl (1:1).

Growth curves of *S. mutans* cultures under PA-compounds treatment were recorded by automatic measurement of OD_600_ each 20 min during 16.5 h, and the results obtained are displayed in Fig. 6. Ten-fold serial dilutions (10^−1^–10^−4^) were obtained from an initial culture at OD_600_ 0.2. Diluted samples were growth under constant phytate compounds treatment at a fixed final concentration (50 µg/mL), due to the limited solubility of phytate complexes (Fig. 6A–D). The profile of the growth curves obtained for 10^−1^ diluted samples from cultures treated with different PA-compounds did not show any significative difference when compare to the untreated control sample (Fig. 6A). Nevertheless, we detected an increase in the duration of the lag phase in cultures treated with PA compounds at dilution factors 10^−2^–10^−4^ (Fig. 6B–D). Precisely, the most significant effects on the lag phase of growth were observed for cultures treated with ZnPhy and SrPhy complexes (Fig. 6D). These results could suggest a bacteriostatic effect of the PA-compounds based on their ability to increasing the time needed for bacterial population to adapt to a new environment as reported^57,58^. Interestingly, SrPhy and ZnPhy treated samples also exhibited this effect when 10^−2^ and 10^−3^ sample dilutions were tested, which suppose an enhancement of the intrinsic bacteriostatic properties registered for PA.

Bacteria induce the biofilm formation in response to environmental signals. The processes by which *S. mutans* undergoes the formation of biofilms are highly conditioned by the quorum sensing (QS) system^59^. QS is activated in response to the release of autoinducer molecules or pheromones in a cell density-dependent manner and confers a bacteria population the ability to alter their physiology and behaviour as a group unit instead of single entities^59,60^. In this sense, QS enables a collective response of bacterial populations when they are exposed to any environmental stress by the regulation of different physiological processes including sporulation, antibiotic production, competence development and biofilm differentiation, among others^60,61^. In our experiments, phytate supplementation altered the levels of biofilm production and the number of viable bacteria embedded in polymeric biofilm, perhaps associated by their role in the modulation of the QS transduction system. In fact, the significantly decreased of CFU found in the biofilm of ZnPhy samples (Supplementary Fig S2) is in accordance to its higher biofilm disaggregation ability (Fig. 5). Furthermore, it could be speculated that the higher OD_600_ values obtained for ZnPhy in Fig. 6 are tentatively attributed to the synergic role between the cation and phytate in *S. mutans* metabolism, which drives the proliferation of viable CFU to the planktonic solution since biofilm formation is expected to be unfavoured as was highly inhibited in Fig. 5. This work demonstrates the possibilities of applying these type of formulations in cariogenic prevention strategies. In fact, the inhibition of the proliferation of key strains such as *S. mutants* in dental plaque, supports further validation for testing these compounds in vivo.

## Conclusions

Two metallic phytate-complexes bearing Sr^2+^ and Zn^2+^ (SrPhy and ZnPhy) have been successfully prepared in high yields. Their deep compositional analysis in the solid-state by spectroscopic techniques (ICP, EDS, Raman, ATR-FTIR, solid ^13^C NMR and ^31^P NMR) along with thermal degradation evaluation, confirmed the metal coordination bond and allowed to define their molecular formula as Sr_4_C_6_H_10_O_24_P_6_·5H_2_O and Zn_6_C_6_H_6_O_24_P_6_·6H_2_O, in a 5-axial/1-equatorial conformation. In vitro bactericidal studies results provided evidences about the capacity of phytate complexes for modulating the intrinsic antimicrobial properties of PA in terms of biofilm disruption and growth trend of *S. mutans*. The highest anti-biofilm activity was exhibited by ZnPhy, followed by SrPhy. This synergic effect between PA and the corresponding cation might affect other metabolic processes, and thus, is of special interest for the evaluation of their biological properties in other aspects. In general, findings of this work envision the potential of the two bioactive metallic complexes to be applied in the biotechnological and biomedical fields.

## Methods

### Synthesis of metallic phytate derivatives

Phytic acid sodium salt hydrate (C_6_H_18_O_24_P_6_.xNa.yH_2_O) was purchased from Sigma-Aldrich, strontium chloride hexahydrate (SrCl_2._6H_2_O) from Acros Organics and zinc chloride (ZnCl_2_ anhydrous) from Fluka. All were used as received without further purification. SrPhy and ZnPhy derivatives were synthesized by reaction of PA with the corresponding metal chloride salt, SrCl_2_ or ZnCl_2_ respectively, following an adapted method from Fernandez-Villa et al.^31^. Briefly, an aqueous solution of phytic acid (0.2 M, pH adjusted to 7.4 with NaOH 0.1 M) was heated at 50 °C in a round bottom flask connected to a reflux system for 2 h. Then, 25 mL of SrCl_2_.6H_2_O or ZnCl_2_ solution (1.2 M, ethanol/water, 1:1, v:v) was dropped onto the previous PA solution in a 1:6 phytic acid:metal (P:M^2+^) molar ratio, forming a white precipitate. The reaction was further stirred for 1 h and quenched down in an ice bath. The solid formed was collected and purified by two hot vacuum-filtrations with a mixture of ethanol/water (300 mL, 1:1, v:v). Finally, the product was dried under vacuum at 50 °C until constant weight and milled to a fine white powder. All compounds were stored at room temperature under anhydrous conditions. The reaction yields were 90% and 96% for SrPhy and ZnPhy respectively.

### Physic-chemical characterization methodologies

Before all the analysis, samples were dried at 50 °C and kept in a desiccator until the HR-TGA analysis, in order to prevent the uptake of water from ambient humidity. The atomic composition of phytate complexes was determined by emission spectroscopy analysis using an inductively coupled plasma (ICP) optical emission Perkin-Elmer 430DV. A given weight of the complex was dissolved in HCl (2% p/v) and the solution was made to volume in a measuring flask. The experimental water content for each molecular formula was obtained by high resolution thermogravimetric analysis (HRES-TGA) using a TGA Q500 apparatus from TA instruments under a inert nitrogen atmosphere or air at a heating rate of 10 °C/min in a range of 40 – 700 °C. Likewise, the chemical composition of phytate complexes was determined by energy-dispersive X-ray spectroscopy (EDS) using a Hitachi SU8000 equipment and elemental microanalysis using a Eurovector EA 3000 equipment.

The structural characterization of the compounds was analyzed by Attenuated Total Reflection Fourier Transform Infrared (ATR-FTIR) spectroscopy (Perkin- Elmer Spectrum One spectrophotometer); Raman spectroscopy (Renishaw inVia Raman Microscope, laser wavelength 785 nm, objective 100 × 0.85 and spectral resolution of 1200 lines/mm); and solid ^13^C and ^31^P nuclear magnetic resonance (NMR) (Bruker AV-400-WB with 4 mm triple channel probe with ZrO rotors, Kel-F plug at room temperature, working frequency 161.97 MHz for ^31^P and 100.32 MHz for ^13^C, and rotation speed 10 kHz in both cases).

### Cell cultures

Biofilm inhibition capacity of phytate compounds was assessed semi-quantitatively by crystal violet (CV, Sigma Aldrich) staining and through the count of viable bacteria (colony forming unit, CFU) found in both the planktonic solution and bacterial biofilm. *S. mutans* CECT 479 was grown in brain heart infusion (BHI, NutriSelect® Plus, Sigma Aldrich) broth medium. Experiments were performed in triplicate with five different inoculums coming from a bacterial solution with an optical density of 0.1 registered at 600 nm (OD_600_ 0.1), recorded in a spectrophotometer model Ultrospec 10 cell density meter (Amersham Biosciences). Bacteria stock inoculum was storage at − 80 °C in 15% (v:v) glycerol solution. PA, ZnPhy and SrPhy were solved at 100 µg/mL in Tris–HCl buffer 50 mM, pH ≈ 7. To do this, the phytate complexes were solved at 2 mg/mL in Tris–HCl buffer 1 M overnight. Subsequently, 5 mL of this solution were diluted in 100 mL of deionised water. The resulting solutions were adjusted to a final concentration of metal complexes of 100 µg/mL, 50 mM of Tris–HCl, and pH close to neutral. Finally, samples were sterilised by filtration with a 0.22 nylon filters. The control samples of these experiments was a *S. mutans* culture in BHI:Tris–HCl buffer 50 mM (1:1) without phytate compounds treatment.

### Biofilm inhibition and viable bacteria

For antimicrobial assays, 10 µL of bacteria solution OD_600_ 0.1 (2.0 × 10^7^ CFU/mL) were inoculated in 10 mL of BHI broth and were cultured aerobically at 37 °C in static conditions until OD_600_ ≈ 0.6 (1.2 × 10^8^ CFU/mL). Then, bacteria were diluted 1:50 in BHI medium and 100 µL of the latter dilution were placed in a flat bottom 96-well plate. Subsequently, 100 µL of the corresponding phytate (100 µg/mL) or control solutions were added to each well and the plate was incubated in static conditions for 5 h at 37 °C. For the quantification of viable cells, 20 µL aliquots were extracted from the supernatant and serial dilutions in BHI medium were prepared to range 10^−1^–10^−6^. Then, the rest of the planktonic solution phase was carefully removed and the biofilm deposited on the wells was disrupted by scratching in a 200 µL solution mixture of BHI:Tris–HCl (1:1). Ten-fold serial dilutions (10^−1^–10^−6^) of these bacterial suspension were prepared. The CFU number, from both the resuspended biofilm and the planktonic, were determined by depositing 10 µL droplets of the diluted bacterial solutions (10^−3^–10^−6^) on Agar-BHI plates, followed by incubation overnight at 37 °C. On its turned, for the semi-quantification of the biofilm produced, after incubation time the supernatant was removed and 50 µL of CV solution (1% w:w) were added to each well and the plate was left to react at room temperature for 15 min. Subsequently, the dye was removed and the wells were washed three times with 200 µL of deionised water. Finally, the remaining stain was solved in 200 µL of ethanol 96% and the absorbance of the samples was measured at 595 nm (A_595_) in a VersaMax microplate absorbance reader (Molecular Devices, USA). Wells without bacteria were stained and washed following the same procedure to establish the basal levels of the dye retained in the walls. Data were expressed as the optical density.

Results were statistically analysed by an ANOVA test between all groups evaluated at significant levels **p < 0.005 and ***p < 0.001 (Tukey Test).

### Determination of growth curves

The growth profiles of *S. mutans* cultures under phytate treatment were assessed spectroscopically by measuring OD_600_ over time. *S. mutans* was grew in BHI medium until OD_600_ 0.2 (5.3 × 10^7^ CFU/mL). Serial dilutions of this bacterial solution were prepared from 10^–1^ to 10^−4^, using a ten-fold dilution factor, and 100 µL aliquots were placed in a 96-well plate along with 100 µL of the corresponding phytate compound (100 µg/mL) or control solution (BHI:Tris–HCl, 1:1). The plate was incubated at 37 °C and shaken for 5 s before each measure. OD_600_ was automatically recorded every 20 min for 16.5 h in the VersaMax microplate absorbance reader.

## Supplementary Information

Supplementary Information.

## Data Availability

Materials, data and associated protocols are available to readers without undue qualifications in material transfer agreements.
