# Supplementary material for: A study on Sr/Zn phytate complexes: structural properties and antimicrobial synergistic effects against Streptococcus mutans
Source: Sci Rep. 2022 Nov 23;12:20177. doi: 10.1038/s41598-022-24300-8 (PMC9684506; doi:10.1038/s41598-022-24300-8)
Supplement: Supplementary file 1 — Supplementary Information. [file 41598_2022_24300_MOESM1_ESM.docx]

**A study on Sr/Zn phytate complexes: structural properties and antimicrobial synergistic effects against *Streptococcus mutans*.**

*Gerardo Asensio ^a^, Ana M. Hernández-Arriaga ^b,e^, Marcela Martín del Campo ^a,c^, Auxiliadora M. Prieto ^b,e^, Luis Rojo ^*a,d,e^, Blanca Vázquez-Lasa ^a,d,e^*

^a^ Instituto de Ciencia y Tecnología de Polímeros, (ICTP) – CSIC, Calle Juan de la Cierva, 3, 28006 Madrid.

^b^ Centro de Investigaciones Biológicas - Margarita Salas (CIB-Margarita Salas) – CSIC, C. Ramiro de Maeztu, 9, 28040 Madrid.

^c^ Facultad de Estomatología, Universidad Autónoma de San Luis Potosí, México

^d^ Networking Biomedical Research Centre in Bioengineering, Biomaterials and Nanomedicine (CIBER-BBN)

^e^ Interdisciplinary Platform for Sustainable Plastics towards a Circular Economy-Spanish National Research Council (SusPlast-CSIC), Madrid, Spain

**Supplementary information**

**

**

Figure S1. HRES-TGA diagrams of PA, SrPhy and ZnPhy obtained in air atmosphere.


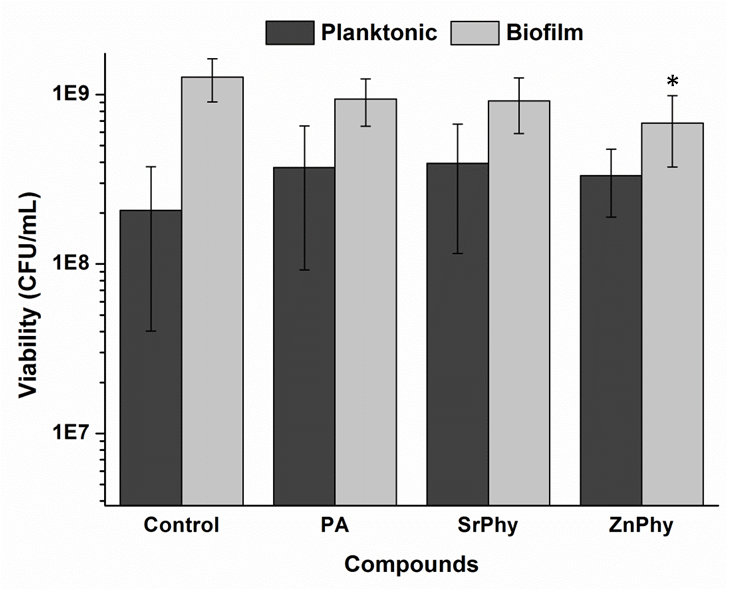


Figure S2. Count of CFU found in the planktonic solution and the biofilm after incubation with phytate compounds (50 µg/mL). The control sample of both experiments was S. mutants growth in BHI:Tris-HCl (1:1). Results obtained for phytate compounds were statistically compared respect to control group by ANOVA test at significant level **p* < 0.005 (Tukey Test).
